# Supplementary material for: ‘‘Mitigating cancer pain: What else matters?”—A qualitative study into the needs and concerns of cancer patients in Sri Lanka
Source: PLoS One. 2025 Jan 24;20(1):e0302615. doi: 10.1371/journal.pone.0302615 (PMC11761611; doi:10.1371/journal.pone.0302615)
Supplement: S1 File — (DOCX) [file pone.0302615.s001.docx]

**Semi-Structured Interview Guide**

**Introduction**

- Welcome and thank the participant and self-introduction, name, and general affiliation
- Explain the general purpose of the interview with the information sheet, and obtained their written informed consent to conduct the interview,
- Explain the presence and purpose of recording equipment.
- Emphasize that there are no right or wrong answers, and participants should feel free to share their honest experiences

**Questions**

1. How does the pain affect your daily life?
   1. Can you explain more about how this problem affects your daily life?
2. Would you please describe your needs related to physical difficulties due to pain?
3. Would you please describe how you could overcome those difficulties
4. Did your pain affect any other aspects of your life
   1. (e.g., Employment, social relationships )
5. How does the pain affect your relationship with your partner and family / How do your family and friends react to your pain
6. How does pain affect your relationships with your friends and other people in your neighborhood
7. Can you describe your needs related to social difficulties due to pain
8. Are there times when you get upset about your pain?
   1. Can you please tell me what you do/feel during such times?
9. With whom would you like to talk/share your concerns
10. Can you describe your needs related to psychological difficulties due to pain
11. To what extent have you received any spiritual support according to your religion?
    1. If yes, what type of support do you receive
12. Do your practices or believe any other non-medical therapies to alleviate pain
13. What are your plans or hopes in relation to pain relief
14. How much satisfied are you with yourself
15. Can you describe your needs related to the spiritual aspect due to pain
16. Do you have enough information about your pain and treatment?
17. How well do you think your health has to be in the future
18. Are there any needs do you have related to pain
19. Is there anything else you would like to share
