# Supplementary material for: ‘‘Mitigating cancer pain: What else matters?”—A qualitative study into the needs and concerns of cancer patients in Sri Lanka
Source: PLoS One. 2025 Jan 24;20(1):e0302615. doi: 10.1371/journal.pone.0302615 (PMC11761611; doi:10.1371/journal.pone.0302615)
Supplement: Attachment — Submitted filename: Response to Reviewers.docx [file pone.0302615.s003.docx]

Dr. NP Edirisinghe,

Senior Lecturer

Department of Fundamental Nursing,

Faculty of Nursing, University of Colombo, Sri Lanka

19.07.2024

Academic Editor and Reviewers

PLOS ONE

**Submission of revised version of original research paper to PLOS ONE**

I would like to express my sincere gratitude for your valuable feedback and constructive comments on my manuscript.

The revisions suggested are tabulated as follows.

**Journal Requirements:**

|  | Comment | Response |
| --- | --- | --- |
| 1. | Please ensure that your manuscript meets PLOS ONE's style requirements, including those for file naming. | Revised the manuscript |
| 2. | Please state what role the funders took in the study. | Added the role of the funder |
| 3. | Please confirm at this time whether or not your submission contains all the raw data required to replicate the results of your study. | Semi-structured Interview guide used to collect data was attached as a supplementary document. |
| **Reviewer #1:** | | |
| 1. | **Abstract:** it would be beneficial to include a sentence on cancer prevalence and trends in Sri Lanka, | Included information on cancer prevalence and trends in Sri Lanka |
| 2. | Introduction: better to give more statistics on the increasing cancer trends in Sri Lanka based on the Cancer Registry data.  it would be beneficial to explicitly connect these statistics to the significance of studying cancer pain specifically in Sri Lanka. | given the statistics on the increasing cancer trends in Sri Lanka and connected the statistics to the significance of studying cancer pain in Sri Lanka |
| 3. | Consider emphasizing the specific challenges faced by Sri Lanka in this context | “A clinical audit found suboptimal pain assessment in cancer patients [7], nurses lack autonomy and specialized knowledge in cancer pain management, often working in a task-oriented system that neglects patients' pain needs [8], In Sri Lanka, limited healthcare resources and inadequate pain management facilities exacerbate these challenges.” |
| 4. | It might help to briefly mention any existing gaps in Sri Lanka's healthcare system that this study aims to address. For example, "Despite the recognized need for holistic care, Sri Lanka's healthcare system faces significant gaps in implementing such approaches." | “Despite advancements in medical therapies, there remains a lack of comprehensive understanding of the essential needs and concerns related to cancer pain, particularly in resource-constrained settings like Sri Lanka. The country's healthcare system, while acknowledging the importance of holistic care, struggles with significant gaps in the implementation of such approaches. This is especially crucial given Sri Lanka's ongoing demographic and epidemiological transition, characterized by an aging population and an increasing prevalence of non-communicable diseases.” |
| 5. | you could briefly outline what specific insights you hope to gain through qualitative research. | “In light of these factors, it is vital to emphasize the significance of conducting this study using a qualitative research approach. Qualitative methods provide unique insights into patients' lived experiences, perspectives, and unmet needs, offering a rich understanding of the complexities surrounding cancer pain. By capturing these nuances, the study seeks to identify patient needs and problems of effective pain management and to understand the emotional and psychological impacts of cancer pain in the Sri Lankan context. Ultimately, these insights will inform the development of more effective, patient-centered interventions tailored to the unique needs of cancer patients in Sri Lanka.” |
| 6. | The objective of the study is stated, but it could be made more prominent. Consider explicitly stating the research question or hypothesis. For example, "This study seeks to explore the primary needs and concerns of cancer patients in Sri Lanka regarding pain management, aiming to inform more effective and patient-centered interventions." | “The study aims to address the challenges associated with cancer pain in Sri Lanka by deepening the understanding of the substantial needs and issues faced by patients. Beyond focusing solely on pain relief, this research will investigate the broader psychosocial concerns that significantly impact the quality of life for cancer patients. These include emotional, psychological, cultural, and social factors that influence patients' experiences and coping mechanisms. By examining these areas, the study seeks to enhance pain recognition and contribute to the development of more effective, patient-centered interventions, ultimately aiming to reduce the overall burden of cancer pain in the country..” |
| 7. | Ensure that the introduction explicitly ties back to the title "Mitigating Cancer Pain: What Else Matters?" by outlining what additional factors (beyond pain relief) are being investigated. For example, "In addition to pain relief, this study will explore the broader psychosocial needs and concerns that impact the quality of life for cancer patients in Sri Lanka." |  |
| **Comments on the Methods Section:** | | |
| 1. | Section 2.1: Study Design The phrase "A qualitative descriptive study was conducted approach to delve into" is grammatically incorrect. Consider revising it to...  The sentence "This study adopts a constructivist perspective, acknowledging the subjective nature of cancer experiencing pain..." is slightly awkward. Consider revising it to "..." | Revised as suggested  “A qualitative descriptive study was conducted to delve into the lived experiences of individual needs and major concerns of patients with cancer pain in Sri Lanka.  This study adopts a constructivist perspective, acknowledging the subjective nature of experiencing cancer pain and seeking to explain and comprehend the many interpretations attributed to these experiences by individuals” |
| 2. | Section 2.2: Study Setting and Participants-  Mention the specific reasons for selecting Apeksha Hospital as the study setting  The process of purposeful selection of study participants is not described adequately  It is unclear whether patients with advanced cancer or palliative patients were included. | Apeksha Hospital is the premier center for cancer care in Sri Lanka.  The process of purposeful selection of study participants is described  Patients fulfilling inclusion and exclusion criteria were included irrespective of advanced cancer and palliative state. |
| 3. | Section 2.3: Study Instruments  Elaborate on the semi-structured interview guide, including whether probing questions were used and what the opening question was / what kind of questions included | Elaborated the content of the semi-structured interview guide, including the use of probing questions and what the opening question was / what kind of questions included. The interview guide was submitted as a supplementary file |
| 4. | Section 2.4: Data Collection  Procedural rigor should be documented by providing information about the researcher’s credentials and previous experience in interviewing and communication to increase reader confidence.  Since more than one interviewer was used, it is important to describe measures taken to maintain uniformity in data collection, such as the training of data collectors.  Consider breaking up the sentence "The interviewer introduced herself and described the purpose of the interview" into two sentences for clarity Consider breaking up the sentence | The sentence “The principal investigator (PI), a registered nurse, and an experienced qualitative researcher conducted semi-structured interviews…” was revised for clarity. The PI was the sole data collector.  “The principal investigator (PI) conducted semi-structured interviews (n=21) until theoretical data saturation was attained with the use of an interview guide. The PI's background in nursing and qualitative research, combined with her previous experience in conducting in-depth interviews and effective communication, ensured the rigorous collection of data and enhanced the reliability of the findings.”  Revised as suggested |
| 5. | Section 2.5: Data Analysis  The description of data analysis lacks detail, particularly regarding the transcription and translation process.  The data analysis section appears incomplete and need more details on analysis is required; Was coding performed on the basis of pre-identified themes or just obtained directly from the interviews? Please specify | Revised the data analysis section with more details. Codes, and themes were obtained from the interviews, there were no pre-identified themes. |
| 6. | Section 2.6: Trustworthiness  This section should be revised to provide a more structured and comprehensive overview of the four components of trustworthiness: credibility, confirmability, dependability, and transferability. | Revised as suggested |
| **Results:** | | |
| 7. | 1st Paragaraph –  Include information on whether participants were working, retired, or had stopped working because of cancer  Time since diagnosis could be categorized further (e.g., 2-3 years, >3 years) to provide a clearer picture of the duration of suffering among included patients. | I added information on the current status of the occupation. There was no recorded information on retired or had stopped working because of cancer.  Time since diagnosis categorized further (e.g., 2-3 years, >3 years). |
| 8. | Table 2. Theme 1: Changing normal lifestyle | The quote "I cannot cook now. I do not eat the way I want to..." is categorized under functional limitations |
| **Discussion** | | |
| 9. | Discrepancy regarding who conducted the interviews | Clarified in the methods section. |
| 10. | The discussion could benefit from further exploration of how the study findings contribute to the existing literature on cancer pain management, particularly in the context of Sri Lanka. | Revised and elaborated the first paragraph of the discussion section. |
| 11. | Expanding the discussion to include potential implications for healthcare practice in Sri Lanka and future research directions | The last two paragraphs added including potential implications for healthcare practice in Sri Lanka and future research |
| **Reviewer #2:** | | |
| 1. | Specify whether participants received any rewards for their involvement in the study | The participants did not receive ant reward for their involvement in the study |
| 2. | Elaborate on how nonverbal responses were utilized in the subsequent analysis | Elaborated on how nonverbal responses were utilized in the subsequent analysis |
| 3. | Describe the measures taken to ensure the quality of data analysis | Described under four components of trustworthiness: credibility, confirmability, dependability, and transferability. |
| 4. | Confirm whether interview transcripts were reviewed by individuals other than the interviewers | The interviewer (PI) reviewed initially and later the members of the research team involved in reviewing. Later two external qualitative research experts were reviewed. |
| 5. | Identify who performed the transcriptions | Transcriptions were done by the interviewer. |
| 6. | Utilize the demographic characteristics collected during the study to enrich the discussion section | Elaborated discussion section including demographic characteristics. |

Thank you for your consideration!

Sincerely,

Nirosha Edirisinghe, PhD

Corresponding author
