# Supplementary material for: ‘‘Mitigating cancer pain: What else matters?”—A qualitative study into the needs and concerns of cancer patients in Sri Lanka
Source: PLoS One. 2025 Jan 24;20(1):e0302615. doi: 10.1371/journal.pone.0302615 (PMC11761611; doi:10.1371/journal.pone.0302615)
Supplement: Attachment — Submitted filename: Response to reviwer.docx [file pone.0302615.s004.docx]

Dr. NP Edirisinghe,

Senior Lecturer

Department of Fundamental Nursing,

Faculty of Nursing, University of Colombo, Sri Lanka

12.12.2024

Academic Editor and Reviewers

PLOS ONE

**Submission of revised version 02 of original research paper to PLOS ONE**

I would like to express my sincere gratitude for your valuable feedback and constructive comments on my manuscript.

The revisions suggested are tabulated as follows.

**Journal Requirements:**

|  | Comment | Response |
| --- | --- | --- |
| 1. | Please review your reference list to ensure that it is complete and correct. If you have cited papers that have been retracted, please include the rationale for doing so in the manuscript text, or remove these references and replace them with relevant current references. Any changes to the reference list should be mentioned in the rebuttal letter that accompanies your revised manuscript. If you need to cite a retracted article, indicate the article’s retracted status in the References list and also include a citation and full reference for the retraction notice. | Reviewed the reference list and revised the 29^th^ reference. |
| **Reviewer #1:** | | |
| 1. | abstract is informative but could benefit from minor refinements to enhance clarity and conciseness | Revised the suggested sentence |
| 2. | as the method of data collection the word “In-depth interviews” are not clearly mentioned in the abstract and methodology section | The data collection method was not ‘In-depth interviews’, it was ‘semi structured interviews. It was mentioned in the abstract and the methodology section. |
| 3. | Administrative Clearance: There is no mention of administrative clearance anywhere in the article. | The sentence on ‘Administrative Clearance’ mentioned under the section 2.4. |
| 4. | Sentence 231 : The term 'multiple sources of data' is misleading, Please revise to accurately reflect the data sources used in this study | Revised enhancing the clarity. |
| 5. | Sentence 262: Consider replacing “salary” with “income” for accuracy. | Replaced “salary” with “income” |
| 6. | Sentence 478: The use of “triangulation” is unclear; if triangulation was not done, please remove this term. | Revised as per the suggestion |
| 7. | Results and Discussion: Aim for a more concise, focused presentation of the results and discussion sections, avoiding repetition. | Some of the repeating contents were removed. Some of the repetition in the manuscript is intentional and serves to emphasize the multifaceted nature of cancer pain, ensuring clarity, depth, and contextual relevance across themes. It reflects recurring patterns in participants' experiences, supporting the validity and trustworthiness of the findings while maintaining their voices at the center of the analysis. |
| **Reviewer #2: No comments** | | |

Thank you for your consideration!

Sincerely,

Nirosha Edirisinghe, PhD

Corresponding author
